# Proteomic Analysis of Plasma Membrane Proteins of Antler Stem Cells Using Label-Free LC–MS/MS

**DOI:** 10.3390/ijms19113477

**Published:** 2018-11-05

**Authors:** Datao Wang, Hengxing Ba, Chenguang Li, Quanmin Zhao, Chunyi Li

**Affiliations:** 1 Institute of Special Wild Economic Animals and Plants, Chinese Academy of Agricultural Sciences, Changchun 130112, China; wangdatao@caas.cn (D.W.); bahengxing@caas.cn (H.B.); lichenguang0916@gmail.com (C.L.); 2 College of Life Sciences, Jilin Agricultural University, Changchun 130118, China; zhaoquanmin6688@gmail.com; 3 Department of Biology, Changchun Sci-Tech University, Changchun 130600, China

**Keywords:** deer antler, antler stem cell, membrane protein, label-free LC–MS/MS, regeneration

## Abstract

Deer antlers are unusual mammalian organs that can fully regenerate after annual shedding. Stem cells resident in the pedicle periosteum (PPCs) provide the main cell source for antler regeneration. Central to various cellular processes are plasma membrane proteins, but the expression of these proteins has not been well documented in antler regeneration. In the present study, plasma membrane proteins of PPCs and facial periosteal cells (FPCs) were analyzed using label-free liquid chromatography–mass spetrometry (LC–MS/MS). A total of 1739 proteins were identified. Of these proteins, 53 were found solely in the PPCs, 100 solely in the FPCs, and 1576 co-existed in both PPCs and FPCs; and 39 were significantly up-regulated in PPCs and 49 up-regulated in FPCs. In total, 226 gene ontology (GO) terms were significantly enriched from the differentially expressed proteins (DEPs). Five clusters of biological processes from these GO terms comprised responses to external stimuli, signal transduction, membrane transport, regulation of tissue regeneration, and protein modification processes. Further studies are required to demonstrate the relevancy of these DEPs in antler stem cell biology and antler regeneration.

## 1. Introduction

The ultimate goal of modern regenerative biology and medicine is to achieve tissue/organ regeneration [1]. Classic models for regeneration studies, such as planaria, zebrafish and salamander, are lower-level animals. Thus, it is not known whether the mechanisms underpinning these regeneration models can be applied to mammals, including human beings. Deer antlers are unusual mammalian organs that can fully regenerate after annual shedding [2,3] and a unique case of stem cell-based mammalian organ regeneration. Morphological and histological studies showed that the distal pedicle periosteum (PP) provides the main cell source for antler regeneration [4,5,6]. PP deletion and membrane insertion experiments demonstrated that PP is the key tissue type that initiates antler regeneration [7]. PP cells (PPC) possess stem cell attributes, such as the markers CD9, CD105, Stro-1, Oct4, Nanog, and SOX2. Rolf, et al. [8] isolated Stro-1+ cells from the PP and antler tip tissues and defined these cells as a type of mesenchymal stem cell. Further characterization of antler stem cells (ASCs) is a prerequisite for advancing our knowledge of antler regeneration.

One key area lacking in understanding of ASCs is our knowledge of proteins associated with the cell membrane in these cells. Membrane proteins represent one-third of the total proteins encoded in the human genome [9]. Due to their interfacial position in cells, plasma membrane proteins play central roles in various cellular processes including signal transduction, cell adhesion, and transport of molecules [10]. While the proteome of ASCs has been studied previously [11,12,13], the majority of differentially expressed proteins (DEPs) and special markers identified were natively soluble proteins. Membrane proteins, characterized by heterogeneity, hydrophobicity, and low abundance, were discarded in these studies since no special enrichment was adopted.

In this study, the plasma membrane proteins of PPCs and deer facial periosteal cells (FPCs) were isolated from their respective tissues and enriched using gradient centrifugation and different solvents. The FPCs were specifically selected to serve as a control as these cells locate in the vicinity of the PPCs and have the most attributes of PPCs, but do not have ability to regenerate antlers [14]. Label-free liquid chromatography-mass spectrometry (LC-MS) was used in this study to detect and analyze the composition of the membrane proteins and the differences between them. Following gene ontology (GO) analysis of DEPs, it was shown that we had successfully enriched plasma membrane proteins. Stem cell surface markers such as CD9, CD29, CD44, CD73, CD90, CD105, and CD166 were detected in the PPCs. GO analysis provided a general profiling for DEPs, and Kyoto Encyclopedia of Genes and Genomes (KEGG) pathway analysis identified some receptors regulated biological pathways in the PPCs. Overall, this study contributes to our understanding of the molecular basis of the stem cells driving antler regeneration.

## 2. Results

### 2.1. Summary of Label-Free Liquid Chromatography–Mass Spetrometry (LC–MS/MS) Results

**Figure 1:**
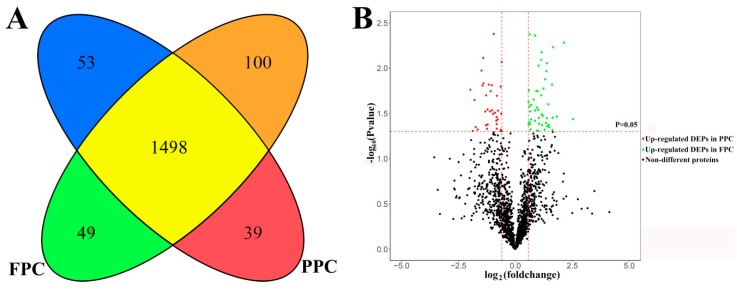
Summary of liquid chromatography–mass spectrometry (LC–MS/MS) results. (**A**) A cartoon showing the intersection of differentially expressed proteins (DEPs) between the pedicle periosteum cells (PPCs) and facial periosteal cells (FPCs), 53 proteins were found solely in the PPCs (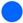) and 100 solely in the FPCs (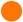). 1498 co-existed in both the PPCs and FPCs without significant difference in expression level (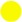), 39 were significantly up-regulated in the PPCs (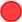) and 49 up-regulated in the FPCs (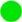). (**B**) A volcano plot displays fold changes and *p*-values of starting MaxQuant data. Fold change >1.5 and *p* < 0.05 were defined as significant difference.

In total, 1739 proteins were identified. Of these proteins, 1586 co-existed in both the PPCs and FPCs; 53 solely in the PPCs and 100 in the FPCs (Supplementary S1). Of the 1586 proteins, 39 were significantly up-regulated in PPCs (*p* < 0.05) and 49 significantly up-regulated (*p* < 0.05) in the FPCs (Figure 1). Therefore, we obtained 241 (53 + 100 + 39 + 49) DEPs in total. All identified proteins were annotated online (available online: http://david.abcc.ncifcrf.gov/, accessed on 9 February 2018), and GO terms containing the key words “plasma membrane” were deemed to be plasma membrane proteins. In the PPCs, plasma membrane proteins accounted for 40.9% (671 out of 1639), and in the FPCs for 40.6% (685 out of 1686). We evaluated the proteomics of PPCs reported by Dong et al. [12] in the same way, and found that 16.3% (407 out of 2500) were plasma membrane protein.

### 2.2. Functional Classification of Differentially Expressed Proteins (DEPs)

**Figure 2:**
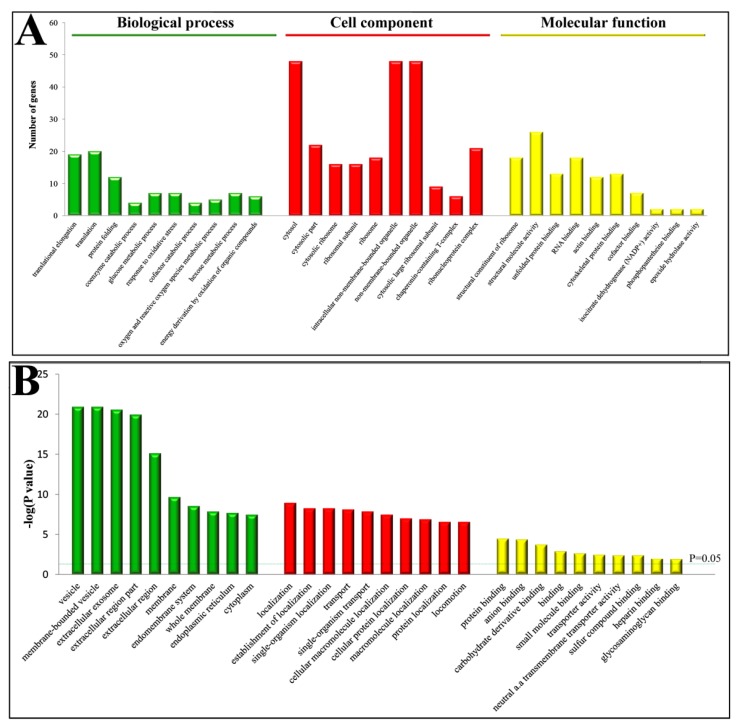
Gene ontology (GO) enrichment analysis of DEPs in the PPCs vs. FPCs. (**A**) Top 10 of each group were involved in “biological processes”, “molecular function” and “cellular component”. (**B**) *p* values of GO terms were indicated.

Gene ontology (GO) annotation and enrichment of DEPs were carried out using online software (available online: http://david.abcc.ncifcrf.gov/, accessed on 9 February 2018). A total of 226 GO terms were significantly enriched (*p* < 0.05). Of these, 124 were involved in “biological processes”, 17 in “molecular function” and 85 in “cellular component” (Supplementary S2). The top 10 GO terms from each category were selected (Figure 2) and showed that the predominant terms in “cell component” were membrane or membrane associated proteins; in “biological processes” were localization and transport proteins; and in “molecular function” were binding proteins. 

**Figure 3:**
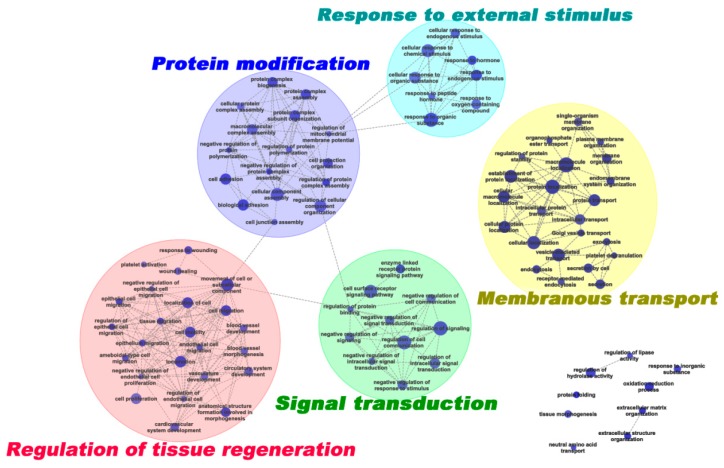
Network of enriched GO terms derived from DEPs. The enriched GO terms are organized as a weighted similar network, with nodes representing enriched GO terms (adj. *p* < 0.05) and edges representing the overlap score (coeffcient cutoff of 0.5), calculated from the number of proteins shared by two GO terms.

The enriched GO terms were further grouped using the EnrichmentMap plusin tool in Cytoscape 3.1.1 [15,16] to generate a weighted similar network (Figure 3). Five representative clusters were generated: (1) in response to external stimulus (8 terms); (2) signal transduction (11 terms); (3) membranous transport (23 terms); (4) regulation of tissue regeneration (24 terms); and (5) protein modification (16 terms).

### 2.3. Interactive Network DEPs

**Figure 4:**
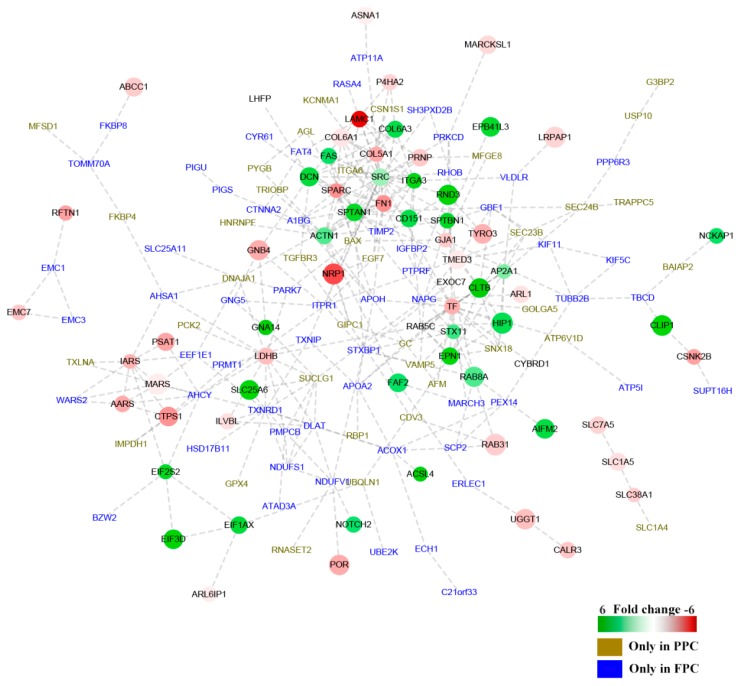
Interaction network of DEPs in the PPCs vs. FPCs. The panorama network consists of 159 DEPs, including 28 up-regulated proteins, 38 down-regulated proteins in the PPCs, 35 solely detected in the PPCs (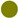) and 58 solely detected in the FPCs (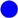). “
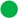
” Denotes up-regulated proteins, and “
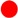
” denotes down-regulated proteins in the PPCs. Bar color was considered as a logarithmic scale from −6.00 to 6.00.

The interactive networks of DEPs were analyzed online (available online: https://string-db.org. version 10.0). One hundred and fifty nine of the 241 DEPs were shown to be involved in the interactive network (28 up-regulated and 38 down-regulated, PPC vs. FPC; 35 solely in the PPCs and 58 in the FPCs), and fold change of proteins was indicated in gradient color (Figure 4). Critical node-proteins in the interactive network were identified following the method mentioned by Boginski and Commander [17]; SPARC (secreted acidic cysteine rich glycoprotein), SRC (Proto-oncogene tyrosine-protein kinase Src), ITGA3 (Integrin alpha-3), TIMP2 (Metalloproteinase inhibitor 2) and TF (Serotransferrin), were identified as critical node-proteins of the network.

### 2.4. Enriched Signaling Pathways

**Figure 5:**
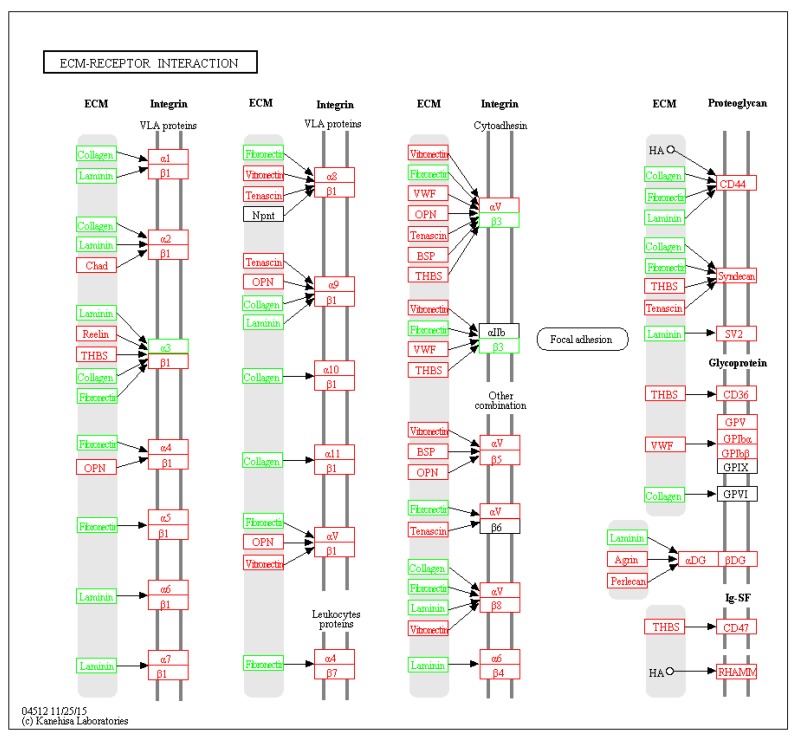
DEPs in the ECM-receptor interaction pathway. Green block: DEPs; red block: genes in transcriptome of the PPCs.

Six pathways of the KEGG were significantly enriched (*p* < 0.05) from the DEPs (Table 1). The extracellular matrix (ECM)-receptor interaction signal pathway significantly dominated and seven related proteins were differentially expressed (Figure 5). For some signal pathways, such as PI3K/AKT and VEGF, although not significantly enriched, key node proteins in the pathways were differentially expressed (Figure S1). Transcriptome data published by our laboratory [12] were used to map whole signal pathways.

### 2.5. Expressed Stem Cell Surface Markers

**Figure 6:**
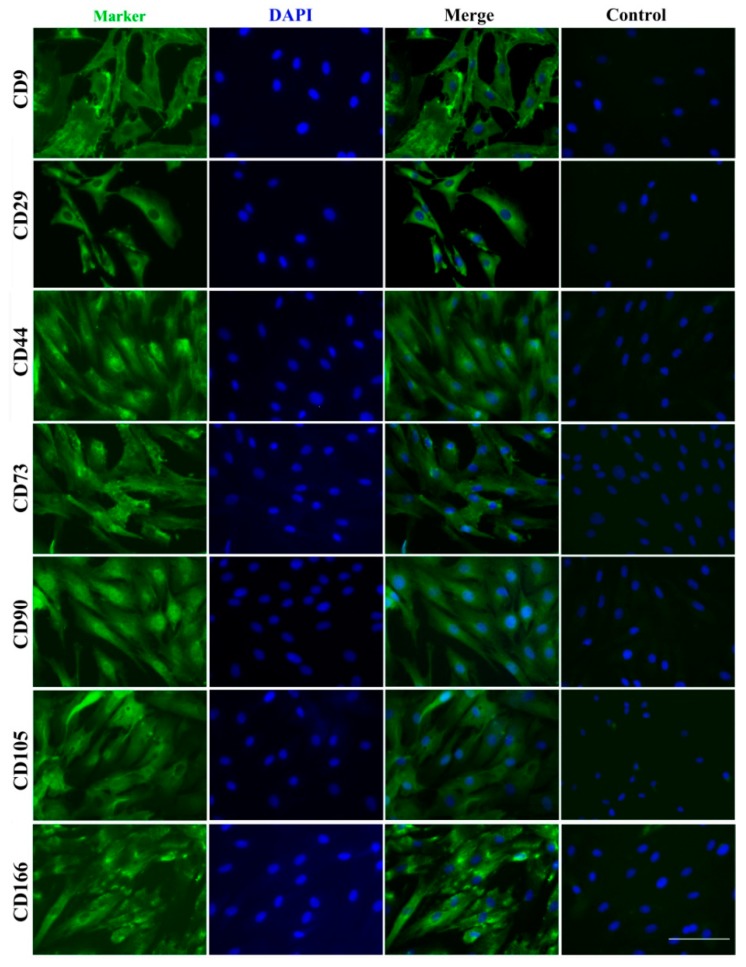
Immunofluorescent staining of surface stem cell markers. CD9, CD29, CD44, CD73, CD90, CD105 and CD166 were detected with their antibodies, and nuclei were counterstained with DAPI. Bar = 100 μm.

In addition to key stem cell membranous surface markers, CD9, CD90 and CD105, reported to be expressed in ASCs [2,8,18], we detected in PPCs, new stem cell markers, CD73, CD90 and CD105 (known as classical markers for mesenchymal stem cells), and CD29, CD44 and CD166 (known as markers for adult stem cells) [19] (Table 2). Immunofluorescent staining further verified the expression of those stem cell surface markers (Figure 6).

### 2.6. Validated DEPs

**Figure 7:**
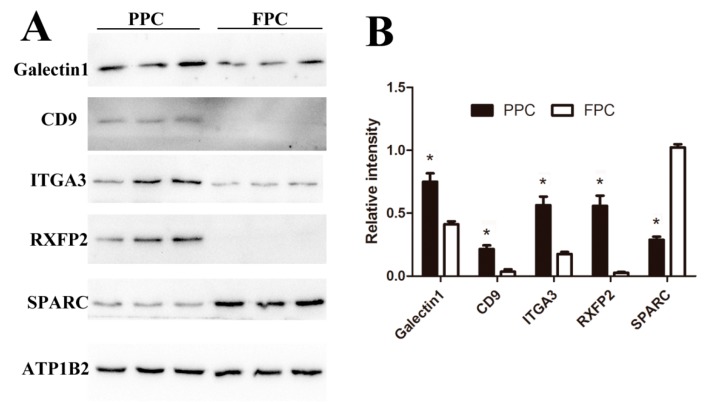
Verification of the DEPs via Western-blot analysis. (**A**) Immunoblot results of five DEPs and internal reference protein (ATP1B2). (**B**) Histogram of immunoblot analyzed using Image J (Version1.8.0, Bethesda, MD, USA) and normalized by ATP1B2. Values are mean ± SD, * *p* < 0.05.

To validate expression levels of our detected DEPs, five proteins (Galectin1, CD9, ITGA3, RXFP2, and SPARC) were confirmed using Western blotting (Figure 7). CD9 and Galectin1 were reported to be expressed in the ASCs previously [13], ITGA3 and SPARC are critical node-proteins from the network (Figure 4), and RXFP2 could be used as the special marker for ASCs [20]. Expressions of Galectin1, CD9 and ITGA3 were found to be up-regulated in the PPCs, while SPARC was down-regulated, and RXFP2 was undetectable in the FPCs (Figure 7A). All five proteins (PPCs vs. FPCs) were highly significantly differentially expressed (*p* < 0.01; Figure 7B). Expression of CD9, ITGA3, RXFP2 and SPARC was consistent with those of LC–MS/MS results. Galectin-1 was found to be overexpressed (2 fold) in the PPCs vs. FPC, but did not reach a significant level (*p* = 0.054). 

## 3. Discussion

Pedicle periosteum cells (PPCs) include “stem cells” that support each annual round of full antler regeneration [2]. This study is the first analysis of plasma membrane proteins of PPCs using label-free LC–MS/MS. A total of 241 significant DEPs were detected in the PPCs vs. FPCs: 92 up-regulated and 149 down-regulated. Among these DEPs, 226 GO terms were identified, comprising six clusters of GO terms involving responses to external stimulus, signal transduction, membrane transport, protein modification, and regulation of tissue regeneration. Furthermore, numerous classical stem cell surface markers were detected, confirming PPCs belong to the stem cell family.

Plasma membrane proteins in cells constitute the sensitive interface between the external and internal environments, and readily react to internal and external stimuli and play central roles in various cellular processes including signal transduction, cell adhesion, and transport of molecules [10]. The fact that plasma membrane proteins accounted for 40.9% of the total proteins in the present study demonstrated that the enrichment process was successful. Although it is impossible to thoroughly purify plasma membrane proteins from organelle and cytoplasmic proteins [21], some non-membrane proteins may be structurally and/or functionally associated with the plasma membrane [22]. In living cells, while many proteins are permanently bound to the lipid bilayer, some proteins, so called “amphitropic proteins”, are temporarily attached either to membrane proteins or to the lipid bilayer [23]. These proteins are recruited to cellular membranes under certain physiological conditions, such as cell signaling and membrane trafficking [24]. In this study, some non-membrane proteins were also isolated together with membrane proteins, indicating that these proteins are closely associated with membrane proteins. SPARC, TIMP2 and FGF7 can bind to membranes temporarily, and we believe that those “amphitropic proteins” are also involved in the control of antler regeneration. 

Due to the nature of our study, i.e. purification of plasma membrane proteins, only six KEGG pathways were found to be significantly enriched from our DEPs, in particular, those of ECM–receptor interaction signaling pathways, which serve important roles in controlling cellular activities such as adhesion, migration, proliferation, differentiation and apoptosis [25]. In this study, ECM–receptor interaction was significantly enriched and seven related proteins were differentially highly expressed (Table 2), suggesting ECM–receptor interaction pathways could be involved in the regulation of antler regeneration. 

Membranous proteins are usually involved at the starting point of signaling pathways, e.g. PI3K/AKT and VEGF signaling pathways, receptors in plasma membrane plays central roles in signal transmission [26,27]. In this study, several key regulatory proteins, such as TGFBR, VEGF, and ITGA/B were significantly up-regulated in the PPCs, indicating that the PI3K/AKT and VEGF signaling pathways are involved in rapid cell proliferation and angiogenesis during antler regeneration. VEGFR2 appears to be the most important receptor in controlling the migration and proliferation of endothelial cells and promoting their survival and vascular permeability [28]. The PI3K/AKT signal pathway regulates fundamental cellular functions such as cell-cycle progression, proliferation, differentiation, and survival [29]. 

TIMP2, down-regulated in the PPCs, is a potent inhibitor of the matrix metalloproteinases (MMP), a group of peptidases involve in ECM degradation. TIMP2 can form a complex with MMP2 to inhibit the activation of MMP2 [30]. Active MMP2 is required for the degradation of ECM, a key physiological process in embryonic development, angiogenesis, reproduction, and tissue remodeling [31,32]. ALCAM, also known as CD166, which is required to activate MMP2 [33], was also detected in the PPCs, suggesting MMP2 is active in the PPCs. Furthermore, ECM–receptor interaction was one of significantly enriched pathways in the present study (Table 2). This pathway controls cellular activities in a direct- or indirect-manner, such as proliferation, differentiation, adhesion, migration, and apoptosis [34,35]. Therefore, the expression of ALCAM and down-regulation of TIMP2 may play important roles in antler regeneration through the ECM–receptor interaction pathway.

The term “Antler stem cells (ASCs)” was put forward to define the cells that give rise to the first and subsequent antlers [4,36] and cells from the antlerogenic periosteum (AP), pedicle periosteum (PP) and reserve mesenchyme (RM) [8,18,37] have been isolated, cultured and partially characterized by several laboratories. CD9 was defined as a cell surface marker of ASCs in previous studies [2,38]. Rolf et al. [8] isolated Stro-1+ cells from the PP, and defined these cells as a type of mesenchymal stem cells (MSCs). CD73, CD90, and CD105 were known as classical markers of MSCs [19]; CD29, CD44 and CD166 were reported as markers for adult stem cells [39]. In this study, a series of stem cell surface markers (CD9, CD29, CD44, CD73, CD90, CD105, and CD166) were found in the membranous proteome of the PPCs, and validated using immunofluorescent staining (Figure 4); therefore, we further confirmed that the PPCs are a type of stem cell.

Male secondary sexual characters of deer antler development are under the control of endocrine factors, especially androgens [40]. Previous studies reported that development of antlers is fully dependent on the changes in androgen and IGF (Insulin-like Growth Factor) levels [2,41]. In vitro studies also show that IGF1 has direct dose-dependent mitogenic effects on the proliferation of antler stem cells (ASCs) [42], whereas, androgens fail to influence the mitogenesis of ASCs [43]. In this study, a cluster containing eight GO terms involving response to external stimuli were enriched, including response to hormones (25 proteins) (Figure 3). Verification of the function of these proteins will help us to reveal the regulatory mechanism of androgens on the initiation of antler formation.

Tissue/organ regeneration is a very complicated process. The liver is unique for its ability to regenerate. More than 100 genes are activated immediately after partial hepatectomy [44] and thousands of genes changed in the expression level within the first hour after partial hepatectomy [45]. EGF, FGF-1, TNF-α, IL-6, and TGF-β play important roles in liver regeneration, however, loss of function of any single gene rarely leads to complete blockage of liver regeneration [46]. Urodele amphibians have the unique ability to regenerate lost limbs. After amputation, cells proximal to the wound surface, including epidermis, dermis, muscle, and cartilage cells, can achieve a multipotential state through dedifferentiation and formed blastema [47]. In the axolotl, de-differentiated cells keep a memory of their tissue origin and re-differentiation back during limb regeneration [48]. Deer antlers are unique in that their regeneration is derived from a single tissue type, i.e. the PP [2]. Li et al. labeled a group of ASCs before antler growth in vivo and found labeled cell progeny in all types of tissues in antlers, including bone, cartilage, dermis and blood vessels [49]. In this study, 24 GO terms involved in the regulation of tissue regeneration were enriched from DEPs in the PPCs vs. FPCs; including wound healing, cell migration, and vasculature development, (Figure 3). We believe that these DEPs or specific regulatory pathways play central roles in ASC biology and antler regeneration. 

## 4. Materials and Methods

### 4.1. Tissue Sampling and Cell Culture

The PP and FP were aseptically collected from 2-year-old male sika deer immediately after slaughtering. Location of the PP and FP are shown in Figure S2. Three biological replicates for both PP and FP were designed, and the corresponding cell lines were created according to the previously reported methodology [14]. All of the above cell types were cultured in Dulbecco modified Eagle medium (DMEM) (Gibco; Grand Island, NY, USA), supplemented with 10% fetal bovine serum (FBS, Gibco), 100 mg/mL of streptomycin, and 100 units/mL of penicillin, and incubated in a humidified atmosphere with 5% CO_2_ at 37 °C. Tissue collection from the slaughtered deer heads in this study was approved by the CAAS Animal Ethics Committee (CAAS2017046C, 22 March 2017).

### 4.2. Plasma Membrane Protein Isolation

Plasma membrane proteins of the PPCs and FPCs were isolated using Minute^TM^ Plasma Membrane Protein Isolation Kit (Invent biotechnologies, Eden Prairie, MN, USA) following the manufacturer’s procedure. Briefly, three 100-mm culture dishes of cells (about 3 × 10^6^) for each sample were harvested using a sterile cell scraper, and washed twice with cold phosphate buffered saline (PBS). The cell pellet was resuspended with buffer A containing protease inhibitors, and subjected to 10 cycles of sonication (5 s on/5 s off). Cell suspensions were transferred to filter cartridges and centrifuged at 16,000 *g* for 30 s. The cell pellet was resuspended again and nucleus (700× *g*, 1 min), cytosol fraction and membrane protein (16,000× *g*, 30 min) were successively separated through gradient centrifugation. Total membrane proteins were resuspended with buffer B, organelle membranes (7800× g, 20 min) and plasma membranes (16,000× *g*, 20 min) were separated once more by gradient centrifugation. The pellet of plasma membrane proteins was dissolved in 200 μL Protein Extraction Buffer.

### 4.3. Protein Identification and Quantification Using LC–MS/MS 

LC–MS/MS analysis was carried out as described elsewhere [50]. Briefly, the proteins (100 μg of each sample) were digested with 3 μg trypsin (Promega, Fitchburg, MA, USA) in 40 μL 25 mm NH_4_HCO_3_ at 37 °C overnight. The concentration of peptides was estimated by MicroBCA assay (Thermo, Rockford, IL, USA). The peptides of each sample were separated on the UltiMate 3000 RSLCnano high-performance liquid chromatography (HPLC) system using a C18 BEH column and C18 Acclaim PepMap RSLC column (Thermo). Peptides were subsequently separated in a linear gradient acetonitrile (from 4% to 35% with 0.1% formic acid) over 140 min and 35–45% over 10 min. At 160 min, the gradient increased to 95% for 10 min. Peptides eluting from the column were analyzed by MS/MS using a Thermo Scientific Q Exactive Orbitrap mass spectrometer (Thermo). MS scans were set at a resolution of 70,000 and 17,500 for the data-dependent MS/MS scans. The MS scan range was from 300 to 1800 *m*/*z*. The peptide signals were then mapped across multiple LC–MS measurements using their coordinates on the mass-to-charge and retention-time dimensions. The total ion current of the peptide signal was then integrated and used as a quantitative measurement of the original peptide concentration. The MS data were analyzed using MaxQuant software version1.5.3.17 (Max Planck Institute of Biochemistryin, Martinsried, Germany) [51], and the parameters were set up following Table 3. Statistical analysis was performed with the *t*-test using SAS (Statistical Analysis System) version 9.0. Fold change > 1.5 and *p* < 0.05 were defined as significantly different in this study.

### 4.4. Bioinformatics Analysis of DEPs

The GO annotation and enrichment of DEPs was analyzed based on the mainstream database David 6.7 (http://david.abcc.ncifcrf.gov/). The PANTHER (protein annotation through evolutionary relationship) classification system (http://www.pantherdb.org/) was used to analyze the molecular characterization of differential proteins. GO results were further clustered using Enrichment Map plugin [16] in Cytoscape 3.1.1 with an overlap coefficient cutoff of 0.5 to generate a network map. The protein–protein interaction of differential proteins was conducted on the String (http://string-de.org/, version 10.0). The differential proteins were mapped to the KEGG database (https://www.kegg.jp/kegg/pathway.html) to enrich KEGG pathways.

### 4.5. Western Blotting

Plasma membrane proteins of the PPCs and FPCs were isolated as previously stated (2.2). Dissolved proteins were separated by 15% sodium dodecyl sulphate-polyacrylamide gel electrophoresis (SDS-PAGE) (20 μg/lane) and transferred to polyvinylidene fluoride (PVDF) membranes. Membranes were blocked with 5% (*w*/*v*) bovine serum albumin (BSA) and immunoblotted with suitably diluted primary antibody followed by secondary antibodies (goat anti-rabbit IgG or anti-mouse IgG) conjugated with horse radish peroxidase. Bands were visualized using chemiluminescence detection reagents (Thermo) applied to autoradiograph films. The quantification of western blot bands was carried out using ImageJ software (version 2.1) normalized by internal reference. All primary antibodies used in this study are listed in Table 4.

### 4.6. Immunofluorescent Staining

Immunofluorescent staining was carried out as described elsewhere [52]. Briefly, cells were seeded on sterile glass cover slips in 24-well plates a day before. Cover slips with adhered cells were rinsed with PBS and fixed in 4% formaldehyde for 20 min. Cells were washed with PBS three times, and blocked with 5% normal goat serum in PBS for 30 min. Cells were incubated with diluted primary antibody overnight at 4 °C, and isotype-matched rabbit or mouse IgG served as the negative controls. Cells were rinsed three times in PBS and incubated with fluorescein-conjugated secondary antibody for 1 hour at room temperature (RT) in the dark. The nuclei of cells were counterstained with DAPI for 5 min at RT. Following washing, the cover slips were mounted on glass slides with anti-fade reagent and examined under a fluorescent microscope.

## 5. Conclusions

Overall, this is the first isolation and analysis of the plasma membrane and associated proteins of ASCs. In this study, we have identified a large number of membrane proteins specifically expressed in the PPCs and stem cell surface markers using the technique of label-free LC–MS/MS. Identified DEPs enriched a set of GO terms impacting various biological processes. Further studies are required to demonstrate the relevance of these proteins and GO terms in ASC biology and antler regeneration.

## Figures and Tables

**Table 1:** Kyoto Encyclopedia of Genes and Genomes (KEGG) pathways enriched in the PPCs vs. FPCs.

Pathway Description	Protein Count	*p* Value	Matching Proteins (Labels), “↑” Up-Regulation, “↓” Down-Regulation
Extracellular matrix (ECM)-receptor interaction	7	0.00882	COL5A1↓, COL6A1↓, COL6A3↑, FN1↓, ITGA3↑, ITGA6↑, LAMC1↓
Adherens junction	6	0.0111	ACTN1↑, BAIAP2↑, CTNNA2↓, PTPRF↓, PTPRM↓, SRC↑
Arrhythmogenic right ventricular cardiomyopathy	6	0.0111	ACTN1↑, CTNNA2↓, DSG2↑, GJA1↓, ITGA3↑, ITGA6↑
Protein processing in endoplasmic reticulum	8	0.018	BAX↑, DNAJA1↑, ERLEC1↑, ERP29↓, SEC23B↑, SEC24B↑, UBQLN1↑, UGGT1↓
Focal adhesion	9	0.018	ACTN1↑, COL5A1↓, COL6A1↓, COL6A3↑, FN1↓, ITGA3↑, ITGA6↑, LAMC1↓, SRC↑
Aminoacyl-tRNA biosynthesis	4	0.0499	AARS↓, IARS↓, MARS↓, WARS2↓

**Table 2:** Expression of stem cell surface markers.

Gene/Protein	Protein IDs	Average LFQ (Label-Free Quantitation) Intensity
CD9 (MRP1)	P30932	3.24 × 10^8^
CD29 (ITGB1)	P53712	3.36 × 10^9^
CD44 (HCAM)	L8ITJ7	1.45 × 10^8^
CD73 (NT5E)	Q05927	6.20 × 10^8^
CD90 (THY1)	L8IGG9	9.42 × 10^8^
CD105 (ENG)	E1B7I8	5.164 × 10^7^
CD166 (ALCAM)	F1MHN8	4.75 × 10^8^

**Table 3:** Parameters setting in MaxQuant comparative analysis.

Item	Value
Enzyme	Trypsin
Max Missed Cleavages	2
Main search	6 ppm
First search	20 ppm
Mass spectrometry (MS/MS) Tolerance	20 ppm
Fixed modifications	Carbamidomethyl (C)
Variable modifications	Oxidation (M), Acetyl (Protein N-term)
Database	uniprot-Pecora_94642_20170405
Database pattern	Reverse
Peptide FDR (False Discovery Rate)	≤0.01
Protein FDR	≤0.01
Time window (match between runs)	2min
Protein Quantification	Razor and unique peptides were used for protein quantification.
LFQ	True
LFQ min. ratio count	1

**Table 4:** Antibodies used in this study.

Terms	Manufacturer and Product Code	Isotype	Application
CD9	LSBio; LS-C46004	Mouse, IgG2	WB ^1^ 1:500; IF ^2^ 1:100
CD29	Proteintech;12594-1	Rabbit, IgG	IF 1:100
CD44	Proteintech; 15675-1	Rabbit, IgG	IF 1:200
CD73	Santa; sc-25603	Rabbit, IgG	IF 1:500
CD90	Bioss; bs-0778R	Rabbit, IgG	IF 1:100
CD105	Elabscience; ESH135	Mouse, IgG1	IF 1:500
CD166	Bioss; bs-1251R	Rabbit, IgG	IF 1:100
Galectin-1	Self-produced	Rabbit, serum	WB 1:100
SPARC	Proteintech; 15274-1	Rabbit, IgG	WB 1:500
ITGA3	Bioss; bs-6328R	Rabbit, IgG	WB 1:200
ATP1B2	Bioss; bs-23414R	Rabbit, IgG	WB 1:500
RXFP2	Self-produced	Rabbit, serum	WB 1:200
Rabbit IgG-Isotype control	Abcam, ab172730		IF 1:200
Mouse IgG-Isotype control	Abcam, ab37355		IF 1:200
HRP-conjugated goat anti-rabbit IgG (H + L)	Beyotime, A0208		WB 1:2000
HRP-conjugated goat anti-mouse IgG	Beyotime, A0216		WB: 1:2000
Goat anti-rabbit IgG H&L (Alexa Fluor 488)	Abcam, ab150077		IF 1:1000
Goat Anti-mouse IgG H&L (Alexa Fluor 488)	Abcam, 150113		IF 1:1000

^1^ WB Western blot; ^2^ IF Immunofluorescence.

## Supplementary Materials

Supplementary materials can be found at http://www.mdpi.com/1422-0067/19/11/3477/s1.
