# Supplementary material for: Proteomic Analysis of Plasma Membrane Proteins of Antler Stem Cells Using Label-Free LC–MS/MS
Source: Int J Mol Sci. 2018 Nov 5;19(11):3477. doi: 10.3390/ijms19113477 (PMC6275008; doi:10.3390/ijms19113477)
Supplement: Supplementary file 1 [file ijms-19-03477-s001.zip › Figure S1-S2.pdf]

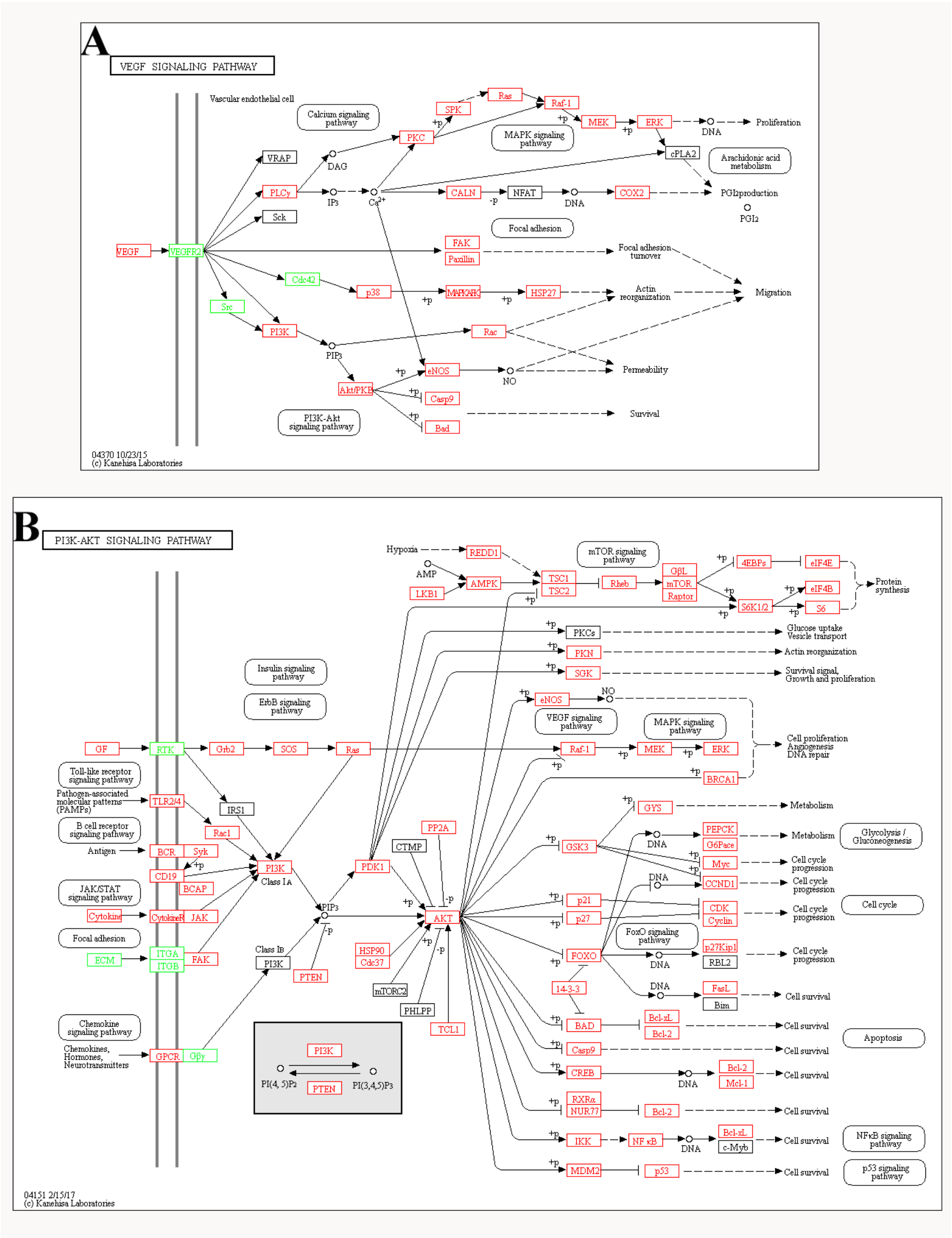

Figure S1. DEPs in VEGF signaling pathway (A) and PI3K-AKT signaling pathway (B). Block in green: DEPs; in red: gene detected in transcriptome of PPCs.

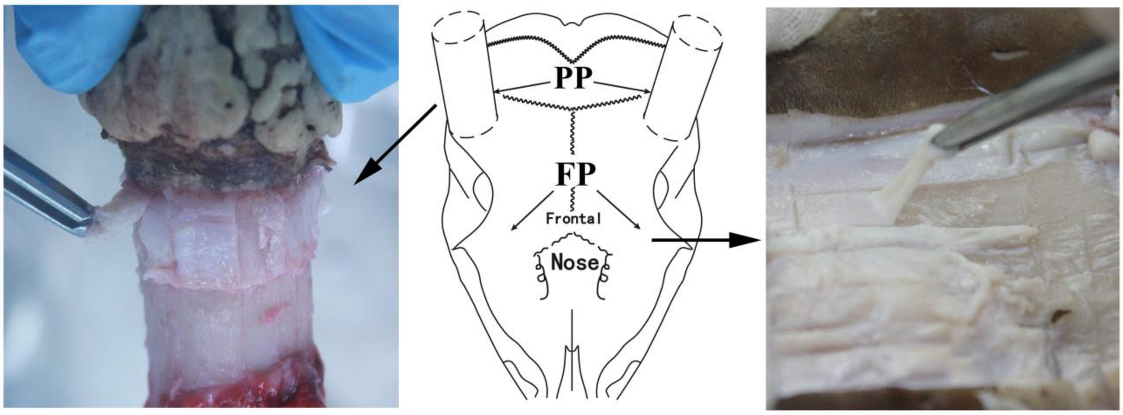

Figure S2. Illustration of tissue location. PP: pedicle periosteum, the tissue which gives rise to regenerating antlers. FP: facial periosteum, the tissue that is nearby the PP but does not have capacity to regenerate antler;
